# Depletion of Tumor-Associated Macrophages Slows the Growth of Chemically Induced Mouse Lung Adenocarcinomas

**DOI:** 10.3389/fimmu.2014.00587

**Published:** 2014-11-25

**Authors:** Jason M. Fritz, Meredith A. Tennis, David J. Orlicky, Hao Yin, Cynthia Ju, Elizabeth F. Redente, Kevin S. Choo, Taylor A. Staab, Ronald J. Bouchard, Daniel T. Merrick, Alvin M. Malkinson, Lori D. Dwyer-Nield

**Affiliations:** ^1^Department of Pharmaceutical Sciences, Skaggs School of Pharmacy and Pharmaceutical Sciences, University of Colorado Denver, Aurora, CO, USA; ^2^Pulmonary Division, School of Medicine, University of Colorado Denver, Aurora, CO, USA; ^3^Department of Pathology, School of Medicine, University of Colorado Denver, Aurora, CO, USA; ^4^Department of Pediatrics, National Jewish Health, Denver, CO, USA; ^5^Research Division, Eastern Colorado Veterans Administration Medical Center, Denver, CO, USA

**Keywords:** macrophage, programing, lung tumor, clodronate, inflammation

## Abstract

Chronic inflammation is a risk factor for lung cancer, and low-dose aspirin intake reduces lung cancer risk. However, the roles that specific inflammatory cells and their products play in lung carcinogenesis have yet to be fully elucidated. In mice, alveolar macrophage numbers increase as lung tumors progress, and pulmonary macrophage programing changes within 2 weeks of carcinogen exposure. To examine how macrophages specifically affect lung tumor progression, they were depleted in mice bearing urethane-induced lung tumors using clodronate-encapsulated liposomes. Alveolar macrophage populations decreased to ≤50% of control levels after 4–6 weeks of liposomal clodronate treatment. Tumor burden decreased by 50% compared to vehicle treated mice, and tumor cell proliferation, as measured by Ki67 staining, was also attenuated. Pulmonary fluid levels of insulin-like growth factor-I, CXCL1, IL-6, and CCL2 diminished with clodronate liposome treatment. Tumor-associated macrophages expressed markers of both M1 and M2 programing in vehicle and clodronate liposome-treated mice. Mice lacking CCR2 (the receptor for macrophage chemotactic factor CCL2) had comparable numbers of alveolar macrophages and showed no difference in tumor growth rates when compared to similarly treated wild-type mice suggesting that while CCL2 may recruit macrophages to lung tumor microenvironments, redundant pathways can compensate when CCL2/CCR2 signaling is inactivated. Depletion of pulmonary macrophages rather than inhibition of their recruitment may be an advantageous strategy for attenuating lung cancer progression.

## Introduction

Lung cancer is responsible for 29% of all cancer deaths in North America, making it more lethal than breast, colon, prostate, and pancreatic cancer combined (1). Approximately 85% of lung cancer cases are smoking-related (2, 3), and tobacco smoke contains both direct carcinogens and agents that promote the growth of nascent tumors. Non-small cell lung cancer (NSCLC) constitutes >75% of lung cancer cases, with adenocarcinoma (AC) being the most frequently diagnosed subtype, regardless of smoking status (4, 5). Lung cancer has long been associated with chronic inflammatory disease. Limiting chronic inflammation may halt the rapid growth and progression of this disease (6, 7) since long-term, low-dose aspirin use reduces the risk of death from lung AC by 45%. However, patients with non-AC subtypes of lung cancer were not protected by aspirin use, suggesting that chronic inflammation may be uniquely important for AC progression (7). In addition, increased numbers of pulmonary macrophages correlate with poor prognosis in NSCLC patients (8–10), and alveolar macrophage numbers also increase during lung tumor progression in mouse models of AC (11, 12). Macrophage depletion early in tumor formation decreases tumor multiplicity (12, 13) indicating a role for inflammatory cells in tumor development even before increased macrophage numbers are detected. Macrophages have been described as obligate partners for breast cancer metastasis to the lung in animal models, and activation of PPARγ in pulmonary macrophages promotes lung cancer progression and metastasis in a murine orthotopic model (14). Prolonged lung inflammation increases tumor multiplicity by promoting clonal expansion of previously initiated cells (15), and chronic anti-inflammatory drug therapy during chemical promotion decreases tumor multiplicity (13, 16). Chronic inflammation drives lung tumor growth and progression in mouse models and human disease, and alveolar macrophages facilitate much of this effect.

Alveolar macrophages produce numerous epithelial growth factors in response to tissue damage, including insulin-like growth factor-I (IGF-I) (8, 17). IGF-I receptor (IGF-IR) is required for anchorage-independent growth of epithelial cells and has been studied in neoplastic proliferation for over 20 years. IGF-IR inhibitors are an area of interest for lung cancer therapy (17, 18). Macrophage IGF-I production is highly induced in response to environmental insult (19). While resident alveolar macrophages are a likely physiological source of lung IGF-I, this growth factor is undetectable in undifferentiated human peripheral blood monocytes (20–22). Alternative macrophage programing occurs early in lung tumorigenesis, corresponding with elevated IGF-I production (23–26). Consistent with this association of tumor growth and enhanced macrophage IGF-I production, transgenic mice that produce twice as much IGF-I in bronchoalveolar lavage fluid (BALF) compared to wild-type controls develop spontaneous lung hyperplasias and adenomas after 12 months (27). Despite the evidence linking lung inflammation, macrophage function, IGF-I production, and tumor progression, the relationship between macrophage-derived IGF-I and lung tumor cell proliferation *in vivo* has not been fully explored. We previously showed that BALF from lung tumor-bearing mice contains 3.5-times more IGF-I than that from naïve mice, and macrophage-produced IGF-I enhances neoplastic proliferation *in vitro* (26), indicating that macrophage IGF-I production may play a major role in early lung tumor progression.

Macrophages are selective targets for liposomal clodronate-induced apoptosis because they aggressively phagocytize liposomes (28). Their increased expression of phospholipases facilitates rapid release of clodronate from the liposome vehicle into the phagocyte upon liposome engulfment (29–31). When administered intratracheally (IT), liposomes do not enter the systemic circulation and deplete only alveolar macrophages (29, 31). Conversely, liposomes given intravenously (IV) systemically deplete myeloid cells in the bone marrow, liver, spleen, and other tissues, and reduce the number of circulating cells available for recruitment to the lungs (32). Herein, we use a combination of IT and IV administration of clodronate liposomes to deplete macrophages from the lungs of tumor-bearing mice, and measure the resulting changes in lung pathophysiology by assessing primary lung tumor growth, macrophage depletion, programing of remaining macrophages, and BALF cytokine contents.

## Materials and Methods

### Mouse lung tumorigenesis

Male A/J mice (6–8 weeks old) were purchased from Jackson Laboratories (Bar Harbor, ME, USA), maintained on hardwood bedding with a 12-h light/dark cycle, and given Teklad-8640 standard laboratory chow (Harlan Teklad; Madison, WI, USA) and water *ad libitum*. CCR2^+/−^ breeding pairs on a BALB/cJ background were kindly provided by Cara L. Mack, M.D. Department of Pediatrics, School of Medicine, University of Colorado, Anschutz Medical Center. BALB/cJ and CCR2^−/−^ mice were bred in the Center for Comparative Medicine (CCM) at the University of Colorado, Anschutz Medical Center. A/J lung tumors were initiated by a single 1 mg/g body weight intraperitoneal (IP) injection of urethane dissolved in 0.9% NaCl (Alfa Aesar; Heysham, Lancashire, UK) as described previously (33). CCR2^−/−^ and wild-type BALB/cJ mice were given six weekly 1 mg/g IP injections of urethane, a regimen shown to reproducibly induce lung tumors in this moderately resistant strain (34). At the times indicated, mice were euthanized by IP injection of sodium pentobarbital (Sigma Aldrich; St. Louis, MO, USA). All animal procedures were performed in accordance with the National Institutes of Health’s Guide for the Care and Use of Laboratory Animals and were approved by the Institutional Animal Care and Use Committee of the University of Colorado, Anschutz Medical Campus.

### Bronchoalveolar lavage

Primary alveolar macrophages and lung protein exudates were isolated by bronchoalveolar lavage (BAL), as previously described (23, 35). The BALF fractions were separated by centrifugation, BAL cells counted, and inflammatory macrophages, lymphocytes, and neutrophils differentiated by Wright Giemsa (Fisher Scientific) staining (23). BAL cell populations from both naïve and lung tumor-bearing mice were composed predominantly of alveolar macrophages (routinely >95%) (10).

### Macrophage depletion by clodronate-encapsulated liposomes

Dichloromethylene diphosphonate (clodronate, 2.5 g; Sigma) was encapsulated in liposomes formed by a 25:1 w/w ratio of phosphatidylcholine:cholesterol (Sigma) as described (31), and the resulting liposomes resuspended in 4 ml sterile PBS. Only 1–2% of clodronate becomes encapsulated, yielding an estimated dose of 0.7–1.0 mg clodronate per 100 μl of liposome suspension. Saline (vehicle) liposomes and clodronate-encapsulated liposomes were synthesized in parallel <2 weeks before use, stored at 4°C, and gently resuspended immediately before instillation or injection (31). Clodronate liposomes for syngeneic transfer experiments were synthesized or purchased from ClodronateLiposomes.org (The Netherlands).

A/J mice bearing urethane-induced lung tumors were anesthetized by a single 50 μl IP injection containing 100 mg/kg ketamine and 10 mg/kg xylazine (CU Clinical Pharmacy; Aurora, CO, USA). Fifty microliters of vehicle or clodronate-containing liposomes were instilled into the lungs via a ball-tip gavage needle bent to a 30° angle and guided by a rodent laryngoscope (Penn Century, Inc.). Follow-up liposome treatments were administered by IV in all mice starting 2 days after IT instillation (100 μl of vehicle or clodronate liposomes administered IV via the tail vein) and repeated once weekly for 5 weeks. IT administration of clodronate is necessary to deplete resident alveolar macrophages, which are not exposed to IV administration, but recruitment of bone marrow macrophages to replenish the alveolar macrophage population can be prevented by IV clodronate liposome ablation of bone marrow monocytes (31).

### Tissue collection

Plasma was obtained by retro-orbital bleeding with heparin-lined capillary tubes (Fisher Scientific) following administration of terminal anesthesia, and stored at −80°C. Lungs were removed following BAL, the lobes dissociated, and tumors dissected from adjacent uninvolved lung under a dissection microscope, as described (33). Tumor diameters were measured by digital calipers, and tumors pooled in pre-tared microfuge tubes (one tube/mouse) and weighed (tumor burden). Tumor dissection and evaluation were conducted in a blinded fashion.

### Immunohistochemistry, tumor grading, and assessment of proliferation

In a similarly treated group of mice, lungs were perfused via the pulmonary artery with 0.9% NaCl, then gently inflated with formalin through the cannulated trachea for 1 h. Lungs were separated into individual lobes and dissected into 14 similarly sized portions and fixed in formalin overnight (33). Lung pieces were embedded in paraffin and sectioned (4 μm). Sections were processed as previously described, and incubated with anti-Ki67 primary antibody (1:200; Fisher Scientific) (33). Incubation with a biotinylated goat anti-rabbit secondary (1:100; Vector Laboratories) was followed by incubation with horse-radish peroxidase conjugated avidin, detection with 3,3-diaminobenzidine (Vector Laboratories), and counterstain with hematoxylin. Sections were evaluated at 400× magnification under an upright microscope (BX41 Olympus), using Spot Advanced software (v4.0.1) to determine tumor area (33). The Ki-67 staining index was calculated by dividing the number of positively staining cells in each tumor by the corresponding tumor area (Ki-67^+^/cm^2^). This Ki-67 index was averaged per animal and then per group. Serial lung sections were stained with hematoxylin and eosin (Fisher Scientific), and lesions graded as hyperplasia (Hyp), atypical adenomatous hyperplasia (AAH), adenoma (AD), adenoma containing a focus of adenocarcinoma (ADwAC) or AC following the guidelines established by Nikitin et al. (36) and using images found at the digital atlas of virtual histological slides as examples (37). Grading was performed in a blinded fashion by three individuals at a multiheaded microscope and evaluated by a board certified pathologist (Daniel T. Merrick). Particular attention was paid to nuclear morphology, density of the lesion, and vessel involvement. We found that adenomas and AC comprised the majority of lesions in this mouse model, and squamous cell and neuroendocrine carcinomas were not observed.

### Determination of macrophage programing by immunofluorescence

Sections were deparaffinized and rehydrated prior to antigen retrieval as described (23). Tissue sections were then incubated overnight at 4°C with 1:50 dilution of anti-arginase I (ArgI, Santa Cruz Biotechnology) primary antibody followed by 1:1000 dilution of Alexa 568-conjugated anti-goat secondary antibody. A mixture of anti-NOS2 (BD Transduction Labs; 1:50 dilution) and anti-F4/80 (ABD-Serotec; 1:50 dilution) primary antibodies were then applied for 1 h at 37°C, followed by 20 min incubations with Alexa 488-conjugated anti-rabbit and Alexa 680-conjugated anti-rat secondaries. Nuclei were stained with DAPI-containing mounting media (Vector Laboratories). Images were obtained with a digital deconvolution microscopy imaging system attached to a Zeiss Axioplan 2 epi-Fluorescence upright microscope. Macrophages were identified by positive F4/80 staining and morphology. Total pixel counts/macrophage were calculated for ArgI, NOS2, and F4/80 immunofluorescence (~50/animal) using ImageJ software (38), and ArgI and NOS2 values were normalized to F4/80 staining. To confirm ArgI^+^ M2 programing, adjacent sections were subjected to a similar IF protocol substituting an antibody against M2 marker phosphoTyr^641^STAT6 (Cell Signaling, 1:50 dilution) for NOS2. Fluorescence intensity was calculated similarly and phosphoSTAT6/ArgI ratios determined.

### BALF IGF-I and cytokine determination

Insulin-like growth factor-I concentrations in BALF were determined by enzyme-linked immunosorbant assay (ELISA) in a 96-well format as directed (R&D Systems, Inc.). For lung cytokine levels, BALF samples were concentrated 5× by centrifugation in 3 kDa molecular weight cut off YM-3 microcon spin-columns (Millipore), and applied to Quantibody^®^ mouse cytokine array slides (Raybiotech, Inc.). Fluorescent Cy3-equivilent antibody signal was read by the CU Cancer Center Microarray Core Facility using a Perkin Elmer Scan Array Ex glass slide laser scanner (Perkin Elmer). Analytes were quantified by regression of log-transformed data sets against within-run standard curves.

### CCL2/CCR2 involvement in lung tumor progression

Tumorigenic mouse lung epithelial E9 cells (39) were maintained in CMRL media (Invitrogen) supplemented with 10% fetal bovine serum. For syngeneic transplant studies, 1 × 10^6^ log-phase E9 cells were suspended in 100 μl of serum-free CMRL media (Life Technologies) and injected subcutaneously (s.c.) into the shaved right flanks of syngeneic male and female BALB/c (WT) or CCL2 receptor null (CCR2^−/−^) mice, a protocol previously shown to generate rapidly growing tumors in nearly 100% of recipient animals (40) Wild-type and CCR2^−/−^ mice received vehicle or clodronate-encapsulated liposomes by IV injection 1 day prior to tumor inoculation (day −1), and once/week thereafter. Tumor size was determined twice/weekly for 24 days, and tumor volume was calculated using the equation for an elliptical cone (as recommended due to the non-spherical growth pattern of the implants): *V* = (*d*^2^ × *l* × π)/6, in which “*d*” is the smallest diameter, and “*l*” the largest. Tumor-bearing mice were euthanized and flank tumors removed and weighed. The experiment was performed twice, with 5–6 mice/group/repetition. Previous studies showed that both macrophage conditioned media (MΦCM; 1:1 mixture of fresh media with media harvested after 24 h incubation with MH-S murine alveolar macrophage cells) and/or IGF-I stimulated *in vitro* proliferation of cultured mouse lung epithelial cells. To determine if this was also true for E9 cells, subconfluent cultures were incubated with MΦCM or 50 ng/ml IGF-I for 48 h, harvested, and relative cell numbers were compared using CellTiter96^®^ proliferation assays (Promega).

### Statistical analysis

Continuous variables were analyzed by two-way ANOVA with Bonferroni *post hoc* comparison to determine significant differences between groups and account for multiple inter-group comparisons. One-way ANOVA with student Newman–Keuls *post hoc* analysis was used to determine significant differences between three or more groups while Student’s two-tailed independent *t*-test was used when only two groups were compared, with Welch’s correction for unequal variances when appropriate. All statistics including Spearman correlations were performed using Prism 5.0 software (Graphpad; La Jolla, CA, USA). Data are presented as mean ± SEM, unless otherwise indicated. In all analyses, *p* < 0.05 was considered to be statistically significant.

## Results

**Figure 1:**
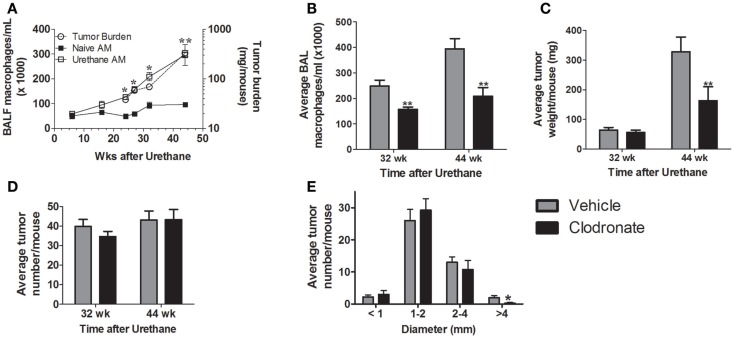
**(A)** Alveolar macrophage numbers increase as a function of tumor growth. Alveolar macrophage (AM) content was determined in naïve (■) and urethane-treated (□) A/J mice as a function of time (left axis). Tumor burden, determined by tumor weight (○, right axis), increased similarly over time (**p < 0.01 tumor-associated vs. naïve alveolar macrophages). Few tumors were detected in naïve mice at any time point (data not shown). **(B–E)** Mice (*n* = 4–6 mice/group) bearing urethane-induced lung tumors were treated with clodronate-containing liposomes (black bars) or the vehicle control (gray bars) for 5 weeks prior to lung harvest at 32 weeks and 44 weeks. **(B)** Alveolar macrophages are depleted by clodronate treatment (**p < 0.01 vs. vehicle) at both 32 and 44 weeks. **(C)** Tumor burden decreases with macrophage depletion at 44 weeks, but not 32 weeks. **(D)** Tumor number remains constant regardless of age or clodronate treatment. **(E)** Tumor multiplicity at 44 weeks broken down by tumor size. Significant difference (*p < 0.05) between treatment groups occurred only in tumors >4 mm in diameter.

**Figure 2:**
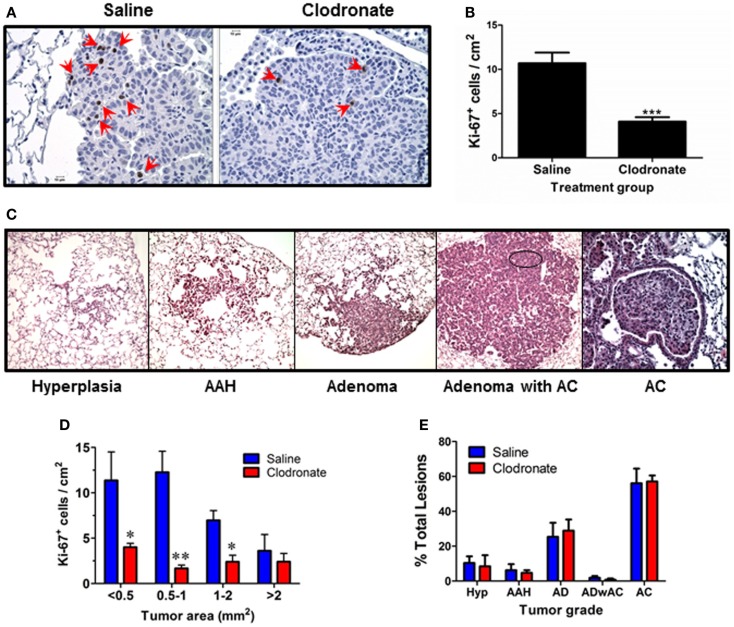
**Effects of clodronate treatment on tumor growth and progression**. **(A)** Representative Ki67 IHC on tissue sections from similar sized tumors from vehicle (left) and clodronate (right) treated mice (400× final magnification). Red arrows point to positively stained tumor cells. **(B)** Ki-67 index was calculated as the average number of Ki-67^+^ cells/cm^2^ tumor area for each tumor in each group (mean ± SEM, **p < 0.01 vs. vehicle). **(C)** Representative examples of each tumor grade (400× final magnification). **(D)** Ki67 index as a function of tumor size at 44 weeks in saline and clodronate-treated mouse lung tumors. Significant differences were seen in the number of Ki67^+^ cells in the smaller lung tumors. **(E)** Percent of each lesion type was calculated/mouse. No significant differences were detected.

Tumor burden and alveolar macrophage numbers (obtained by lavage) increased similarly over time in urethane-treated A/J mice (Figure 1A), while alveolar macrophage numbers changed little over the same time course in naïve mice. Few tumors are detected in naïve mice (data not shown). Liposomal clodronate significantly depleted alveolar macrophages in tumor-bearing mice [harvested 32 (37%) and 44 (48%) weeks after urethane injection compared to vehicle liposome treated, tumor-bearing mice (Figure 1B)]. Tumor burden at the 44-week time point decreased by ~50% with clodronate treatment (Figure 1C) while tumor number did not change (Figure 1D). Comparing tumor weight at 44 weeks to that of mice sacrificed at 32 weeks suggests that tumors did not regress with clodronate treatment, but simply did not grow as rapidly (Figure 1C). A significant decrease in tumors with diameter >4 mm was detected, indicating that clodronate preferentially slowed the growth of larger tumors (Figure 1E). Immunohistochemical staining of Ki67, a marker of cell division, decreased by >50% in tumors from 44-week clodronate-treated mice compared to the vehicle liposome-treated controls (Figures 2A,B), affecting tumors of varying size (Figure 2D). Similar numbers of hyperplasias, AAH, adenomas, adenomas with AC-like foci, and AC (Figures 2C,E) were detected in the lungs of both vehicle and clodronate-treated mice.

**Figure 3:**
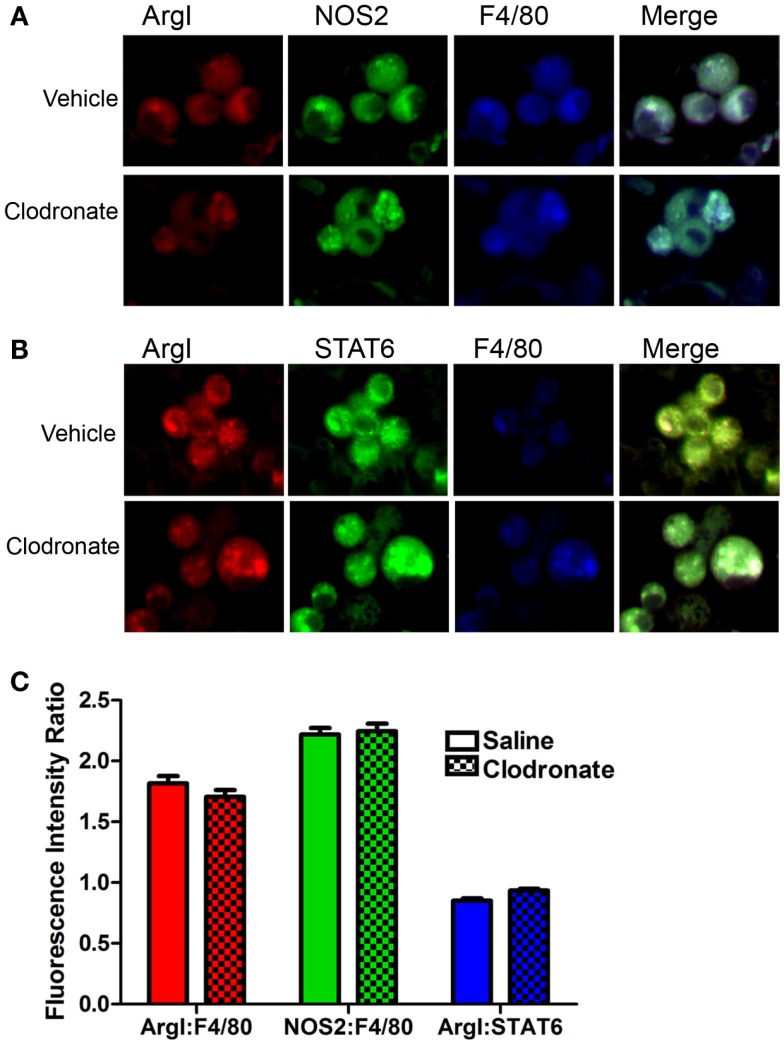
**Immunofluorescent determination of macrophage programing**. **(A)** Alveolar macrophage ArgI (red), NOS2 (green), and F4/80 (blue) expression and the merged image (showing co-localization) in tumor-bearing mice treated with saline or clodronate-containing liposomes. **(B)** ArgI (red), STAT6 (green), and F4/80 (blue) expression and the merged image showing co-localization in tumor-bearing mouse lung treated with saline or clodronate-containing liposomes. **(C)** Pixel counts/macrophage of ArgI (red) or NOS2 (green) normalized to F4/80 (blue) or STAT6 (blue)/ArgI ratios in saline (open bars) or clodronate (cross-hatched) liposome-treated lung (mean ± SEM, *n* = 135–150 macrophages/group).

Programing in tumor-associated macrophages (TAMs) was examined by immunofluorescence to determine whether clodronate liposomes targeted a specific subset of macrophages in mice bearing 44-week lung tumors. TAMs in this study exhibited a mixed M1/M2 phenotype characterized by both NOS2 (an M1 programing marker) and ArgI (an M2 programing marker) expression (Figure 3A). Because earlier studies showed predominantly NOS2^+^ macrophages in this model at this late time point, a second M2 marker, phosphoSTAT6, was analyzed confirming the presence of M2 markers (Figure 3B). Previously, we determined that human lung TAMs express similar mixed (NOS2^+^CD206^+^) programing (23). We saw no large-scale differences in programing marker expression between vehicle and clodronate exposed macrophages (Figure 3C), and ArgI/phosphoSTAT6 ratios were also similar between groups (Figure 3C) suggesting that the TAMs in this model are similarly programed, and this programing is not affected by clodronate exposure.

**Figure 4:**
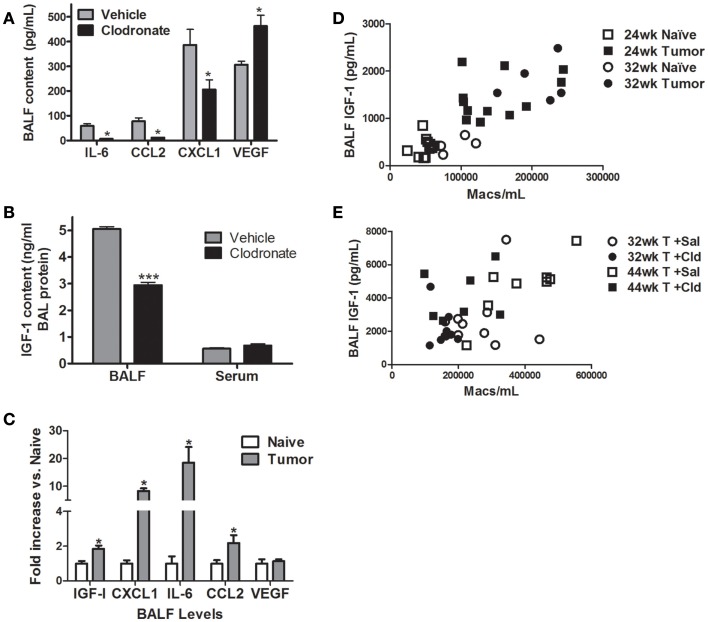
**Effects of macrophage depletion on BAL cytokine content**. Cytokine levels were determined in BALF from naïve (white bars), vehicle liposome (gray bars), or clodronate liposome (black bars) treated mice 44 weeks after urethane treatment. **(A)** Clodronate administration decreased IL-6, CCL2, and CXCL1 in BALF, while VEGF increased 1.5-fold. Data presented as mean ± SEM, *n* = 4–6 mice/group (*p < 0.05 vs. vehicle treated mice). **(B)** BALF IGF-I levels decreased with clodronate treatment in AC-bearing mice (***p < 0.001 vs. vehicle). **(C)** Fold change in BALF IGF-I, IL-6, CXCL1, CCL2, and VEGF levels in age-matched naïve and tumor-bearing mice 32 weeks after urethane exposure (*p < 0.05) **(D)** Correlation between IGF-I and BAL macrophage number in naïve and urethane-treated mice 24 and 32 weeks after urethane treatment (Spearman ρ = 0.8055, *p* < 0.0001). **(E)** Correlation between IGF-I and BAL macrophage number in 44-week urethane mice treated with saline and clodronate liposomes (Spearman ρ = 0.4990, *p* < 0.0031).

Cytokine and IGF-I levels were measured in BALF from vehicle and clodronate-treated, tumor-bearing mice 44 weeks after urethane. BALF levels of GM-CSF, IFN-γ, IL-1α, IL-1β, IL-2, IL-4, IL-5, IL-9, IL-10, IL-12, IL-17, M-CSF, and RANTES were unchanged by clodronate treatment (data not shown). IL-3, IL-13, and TNF-α contents were below the limit of detection in all samples (data not shown). Clodronate treatment significantly decreased levels of IL-6, CCL2, CXCL1, and IGF-I, while VEGF levels increased 1.5-fold (Figures 4A,B). Serum levels of IGF-I did not change with clodronate exposure (Figure 4B). Levels of IGF-I, CXCL1, IL-6, and CCL2 were higher in BALF from tumor-bearing mice than naïve mice 32 weeks after urethane treatment, but VEGF levels were unchanged (Figure 4C). IGF-I levels correlate significantly with BAL macrophage numbers in naïve and tumor-bearing animals (Figure 4D, *p* < 0.0001) as well as in animals exposed to vehicle and clodronate-containing liposomes (Figure 4E, *p* < 0.03). Activated macrophages produce IL-6, CCL2, CXCL1, and IGF-I, but concomitant production of all four signaling molecules also indicates a “mixed phenotype” of macrophages since IL-6 and IL-8 (human ortholog of murine CXCL1) are most often associated with M1 programing and IFG-I and CCL2 with M2 programing.

**Figure 5:**
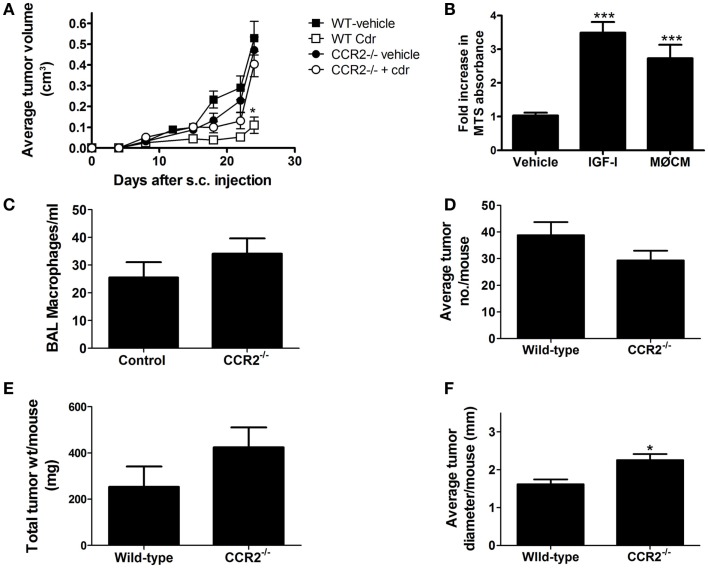
**Clodronate reduces syngeneic implant growth in wild-type (WT), but not CCR2^−/−^ mice**. **(A)** E9 cells were injected into the flank of female BALB/cJ (WT) or CCR2^−/−^ mice. After tumors were established (8 days post injection), clodronate-containing or vehicle liposomes were injected IV once/week until sacrifice. Tumors were measured 2×/week and volumes calculated. Clodronate liposome treatment (Cdr) decreased tumor growth in wild-type mice (*p < 0.05, *n* = 10–12 mice/group from two independent experiments). **(B)** E9 cells were cultured in serum-free media (vehicle), 50 ng/ml IGF-I containing media, or macrophage conditioned media (MΦCM). Cell density was determined by MTS and graphed as fold-increase over control (mean ± SEM, ***p < 0.001 vs. control). **(C–F)** Wild-type BALB/cJ and CCR2^−/−^ mice were injected with 1 mg/g urethane once/week for 6 weeks. BALF and tumors were harvested 42 weeks after the first injection and BAL macrophages (×1000) counted, and lung tumors counted, measured, and weighed (mean ± SEM, *p < 0.05 vs. wild-type, *n* = 5).

Vehicle treated mice secreted 6.5-fold more CCL2 into BALF compared to clodronate-treated mice (Figure 4A). As CCL2 is a chemotactive factor for monocytes and could be integral in the recruitment of macrophages to the site of tumors development, we tested whether ablation of CCL2/CCR2 signaling affected tumor growth in a syngeneic transplant model. Transformed mouse lung epithelial E9 cells were injected into the flanks of immunocompetent syngeneic BALB/cJ wild-type or CCR2^−/−^ mice. After tumors were established, mice were subjected to systemic liposome-based macrophage depletion. There were no significant differences in tumor growth among the vehicle treated wild-type, vehicle treated CCR2^−/−^, or clodronate-treated CCR2^−/−^ mice. However, flank tumors in wild-type mice receiving clodronate liposomes grew significantly slower (Figure 5A). We previously showed that macrophage conditioned media (MΦCM) increases proliferation of mouse lung epithelial cells *in vitro*, and IGF-I is the major component in MΦCM that contributes to this proliferative effect (26). Both MΦCM and IGF-I increased E9 proliferation by more than threefold over control media suggesting that the inhibition of syngeneic tumor growth, resulting from systemic macrophage depletion may be due to decreased macrophage production of IGF-I (Figure 5B).

Ablation of CCL2/CCR2 signaling did not affect the growth of syngeneically transplanted E9 cells, and although there was a slight, but not significant trend of slower growth of the E9 tumors in the CCR2^−/−^ mice, the effect was not as dramatic as that seen with clodronate treatment. In addition, tumor growth in CCR2^−/−^ mice was not significantly slowed by clodronate exposure. To test whether CCL2/CCR2 signaling is required for the *de novo* development of tumors in the lung, wild-type and CCR2^−/−^ mice were initiated with six weekly urethane injections. Lung tumors were harvested, counted, and tumor burden (by weight) assessed at 20, 32, 38, and 42 weeks after the initial urethane exposure. No significant decreases in tumor number, tumor burden, or alveolar macrophage numbers were detected in CCR2^−/−^ mice at any time point (data not shown). Results from the 42-week time point are shown in Figures 5C–F. Not only did CCR2^−/−^ mice show similar number of alveolar macrophages and lung tumors at this late time point, there was a significant increase in lung tumor diameter in the CCR2^−/−^ mice. As CCL2/CCR2 signaling was ablated in the CCR2^−/−^ mice, either a redundant pathway compensates for macrophage recruitment to the lung or resident macrophages proliferate to compensate for the lack of CCL2/CCR2-mediated macrophage recruitment.

## Discussion

Depletion of pulmonary macrophages by nearly 50% decreased the growth of the largest lung ACs in the A/J urethane model. Decreased Ki67 staining indicated that cell division slowed as a result of macrophage depletion *in vivo*, an observation complementing previous results showing that macrophage co-culture and exposure to macrophage conditioned media increased tumor cell proliferation *in vitro* (26). Macrophage depletion did not cause tumor regression as tumor number and stage did not change after clodronate treatment. TAMs produce signals that support tumor growth and promote tumor cell survival. When TAMs are depleted, production of these signals decreases causing a reduction in tumor cell proliferation. Clodronate exposure decreased BALF levels of IGF-I, IL-6, CXCL1, and CCL2 and increased VEGF levels while not affecting most of the other factors examined (largely T_H_1 and T_H_2-associated cytokines), suggesting that alveolar macrophages may not be their primary source in murine lungs. IGF-I involvement in lung tumorigenesis is well established, and the positive correlation of pulmonary BAL IGF-I levels with macrophage numbers (Figures 4C,D) suggests that macrophage production of IGF-I is important in maintaining tumor growth. The decrease in IGF-I levels caused by clodronate-induced macrophage depletion may be partially responsible for slowing lung tumor growth *in vivo*, which is consistent with our previous observations of IGF-I mediation of a significant portion of MΦCM induced lung tumor cell proliferation *in vitro*. The increased BALF VEGF levels detected in the clodronate-treated animals may occur in response to clodronate-induced macrophage apoptosis, as VEGF expression is induced in macrophages that clear apoptotic cells by efferocytosis (41, 42). The remaining healthy alveolar macrophages may express more VEGF as they clear the apoptotic macrophages, resulting from clodronate depletion.

The attenuation of CCL2 production caused by clodronate liposome exposure suggested that monocyte recruitment might also play a role in tumor progression. CCL2 (MCP-1; monocyte chemotactic protein-1) is involved in recruitment of monocytes (43), T cells (44), and dendritic cells (45) to areas of inflammation induced by tissue injury or infection. High levels of CCL2 are associated with poor prognosis in breast cancer (46) and pancreatic cancer (47). However, Zhang et al. (48) found that CCL2 over-expression is associated with improved survival in NSCLC patients indicating that CCL2/CCR2 signaling may have different roles in different tissues. To determine whether CCL2 signaling affected lung tumor growth in mice, syngeneic mouse lung tumor cells were transplanted into wild-type and CCR2^−/−^ mice. Ablation of CCL2/CCR2 signaling had no effect tumor growth, and the lack of CCL2/CCR2 signaling actually nullified the growth inhibitory effects of clodronate liposomes for reasons, which remain to be determined. Because E9 cells are already transformed and do not require a progression phase for tumor growth and because alveolar macrophages differ in function and response from macrophages present near subcutaneous tumors (49), we tested whether CCL2/CCR2 signaling was required for *de novo* lung tumor formation. BAL macrophage content and tumor numbers from urethane-exposed CCR2^−/−^ and wild-type mice were similar between genotypes at each time point. A slight, but significant increase in tumor size in the CCR2^−/−^ mice compared to wild-type littermates was detected at the 44-week time point, which is consistent with the poorer survival observed in human NSCLC patients with low-CCL2 expression (48). Macrophages are known to produce CCL2 in response to IL-4/IL-13 stimulation (50), and the increase in CCL2 production we detected during lung tumorigenesis indicates that there is a Th2 response occurring in lungs during tumor formation. The CCL2 receptor (CCR2) is expressed only in monocyte-lineage cells and T lymphocytes, so CCL2 is unlikely to directly affect neoplastic epithelial cells (9). CCL2 may regulate macrophage recruitment following lung injury, as CCL2 levels in BALF are elevated just prior to the macrophage influx that follows chemically induced pneumotoxicity (51). The similar alveolar macrophage content in tumor-bearing lungs from both wild-type and CCR2^−/−^ mice suggests that either there are redundant macrophage recruitment pathways at play in the lung tumor microenvironment or that the increased number of TAMs results from proliferation of resident macrophages rather than recruitment as has been reported (52).

High-serum IL-6 levels correlate with poor survival and poor response to chemotherapy in NSCLC patients (53, 54). IL-6 is an inflammatory cytokine produced primarily by T cells and M1-programed macrophages to stimulate immune response to injury and trauma. IL-6 may be required for M1 programing in some macrophages, but IL-6 production can induce M2 programing in certain systems to limit existing inflammation (55). Recently, Fernando et al. showed that M2 programing is enhanced by IL-6 exposure in cultured macrophages and suggested that IL-6 augments cytokine expression during both M1 and M2 programing (56). Similar to our findings, Karnevi et al. report that human pancreatic tumor-educated macrophages display mixed M1/M2 programing and produce increased IL-6 and IL-8 (57). Pine et al. report that increased IL-6 and IL-8 production are associated with increased lung cancer risk (58), and IL-6 and IL-8 are both associated with increased NSCLC cell proliferation (59) although neither directly induces increased cell division. Human IL-8 (CXCL8) and murine keratinocyte-derived chemokine CXCL1 (KC) are orthologous in function. CXCL1 production is high in murine alveolar macrophages (60, 61), but not in peritoneal macrophages (62–65). The specific combination of cytokines and surfactant proteins intrinsic to the lung make alveolar macrophages functionally unique compared to macrophages from other tissues. Lung production of IL-8/CXCL1 is induced by inflammatory stimuli through NF-κB and AP-1 activation (63, 65), and both NSCLC cells and macrophages express IL-8/CXCL1. IL-8/CXCL1 is a pro-angiogenic factor as well as a chemotactic factor for neutrophil recruitment to the lungs during emphysema and lung cancer. Few neutrophils were detected in the lungs of vehicle or clodronate liposome-treated tumor-bearing mice (data not shown), so we could not determine whether clodronate treatment altered their numbers further.

Macrophage programing in mouse lung is homogeneous, probably due to the small size of the organ. As urethane-exposed A/J mice form >30 tumors/mouse and these tumors are spread evenly throughout the lobes, alveolar macrophages are exposed to tumor produced factors throughout the lung. The A/J urethane model of lung cancer is unique among most murine lung tumor models in that TAMs surrounding late-stage ACs express high levels of NOS2 and little ArgI, indicating that they are primarily M1-programed (23, 24, 34). However, BALF cytokine levels in these mice indicate that macrophage programing might be more complex. Although ArgI expression decreases as tumors progress, production of certain M2-associated signaling molecules (i.e., IGF-1, CCL2) increases. When these mice were exposed to either vehicle or clodronate-containing liposomes, we detected an increase in both ArgI and phosphoSTAT6 expression (M2 programing markers) in the same macrophages that maintained high-NOS2 expression. The M1/M2 classification represents a continuum of plasticity and does not encompass the functional diversity of macrophages (66), and the measurement of biomarker expression rather than activity and/or function may not yield an accurate picture of macrophage programing. In our study, we detected NOS2^+^ArgI^+^ macrophages, but we did not measure NO levels or determine arginase activity, so although both enzymes were present, we do not know that both were active in the same cells. The presence of phosphoSTAT6 in these TAMs indicates that they were more “M2-like” and the presence of CCR2 and IGF-1 in the BALF supports this. Although there were fewer macrophages in the clodronate-treated lungs, the ratio of ArgI to NOS2 remained constant in the remaining cells indicating that the remaining tumor microenvironment and not macrophage depletion continued to affect the programing of the remaining macrophages.

Our previous studies indicated that TAMs near human lung AC also expressed M1 and M2 markers simultaneously (24), and others have seen similar mixed phenotypes in TAMs in pancreatic (57) and gastric cancer (67). Mixed macrophage programing was also demonstrated in the resolution of liver fibrosis (68) and pulmonary fibrosis (69) and in the early stages of diet induced obesity (70). These mixed populations could be due to catching the macrophages as they change from one programing state to another as in the resolution of a disease, or as they respond to conflicting microenvironmental signals. The detection of both inflammatory and anti-inflammatory cytokines in BALF from these mice indicates that these macrophages do not follow the canonical roles of M1 or M2 macrophages. Further research to determine phagocytic and efferocytic activity, proliferative capacity, and gene expression of TAMs from tumor-bearing mice before and after exposure to vehicle and clodronate liposomes is necessary to characterize this novel macrophage population. The ability of clodronate liposomes to deplete pulmonary macrophages may be enhanced by changing the composition of the liposome delivery vehicle to more effectively and specifically target TAMs that accumulate in the lungs of tumor-bearing mice. These mixed phenotype macrophages may express scavenger receptors such as the mannose receptor, so adding mannose to the liposome surface may increase their uptake, resulting in greater depletion (71). Also, M2 macrophages exhibit enhanced efferocytosis (72) while M1 macrophages are professional phagocytes, so the presence of phosphatidyl serine in the liposome membrane to mimic apoptotic cells and/or lipopolysaccharides on the exterior of the liposome to mimic bacterial cell walls may lead to enhanced uptake and possibly increased macrophage depletion. As IGF-I signaling activates survival factors such as AKT in tumor cells (26), macrophage depletion and the subsequent decrease in BALF IGF-I levels may make tumors more sensitive to therapy-induced death. Although macrophage depletion cannot eliminate lung tumors, it may sensitize tumors to classic chemotherapeutics and radiation by removing both macrophage-produced survival factors from the lung prior to cytotoxic therapies and growth promoting signals such as IGF-I during tumor recovery. Finally, by decreasing the permissive nature of the tumor microenvironment, macrophage depletion may allow the host’s own defenses to recover and eliminate tumor cells.

## Conflict of Interest Statement

The Guest Associate Editor Laurel L. Lenz declares that, despite being affiliated to the same institution as the authors Jason M. Fritz, Meredith A.Tennis, David J. Orlicky, Hao Yin, Cynthia Ju, Daniel T. Merrick, Alvin M. Malkinson and Lori D. Dwyer-Nield, the review process was handled objectively and no conflict of interest exists. The authors declare that the research was conducted in the absence of any commercial or financial relationships that could be construed as a potential conflict of interest.
