# Corrigendum: Depletion of Tumor-Associated Macrophages Slows the Growth of Chemically Induced Mouse Lung Adenocarcinomas

**DOI:** 10.3389/fimmu.2015.00088

**Published:** 2015-03-03

**Authors:** Jason M. Fritz, Meredith A. Tennis, David J. Orlicky, Hao Yin, Cynthia Ju, Elizabeth F. Redente, Kevin S. Choo, Taylor A. Staab, Ronald J. Bouchard, Daniel T. Merrick, Alvin M. Malkinson, Lori D. Dwyer-Nield

**Affiliations:** ^1^Department of Pharmaceutical Sciences, Skaggs School of Pharmacy and Pharmaceutical Sciences, University of Colorado Denver, Aurora, CO, USA; ^2^Pulmonary Division, School of Medicine, University of Colorado Denver, Aurora, CO, USA; ^3^Department of Pathology, School of Medicine, University of Colorado Denver, Aurora, CO, USA; ^4^Department of Pediatrics, National Jewish Health, Denver, CO, USA; ^5^Research Division, Eastern Colorado Veterans Administration Medical Center, Denver, CO, USA

**Keywords:** inflammation, macrophages, programing, M1, M2

In the original manuscript, Hao Yin’s name was misspelled. The correct name is Hao Yin. The incorrect spelling is Hao Lin.

The original article has been updated.

## Conflict of Interest Statement

The authors declare that the research was conducted in the absence of any commercial or financial relationships that could be construed as a potential conflict of interest.

